# Freeze-Dried Mother's Own Milk for Novel Fortification in a Late Preterm Infant with Complicated Intestinal Atresia and Congenital Shortened Bowel: A Case Report

**DOI:** 10.1055/a-2832-1849

**Published:** 2026-03-24

**Authors:** Allyson Ward, Sarah M. Reyes, Berkley Luck, Laura Serke, Lauren Figard, Amy Kimball

**Affiliations:** 1 UChicago Medicine AdventHealth, Division of Women and Children's, Hinsdale, Illinois, United States; 2 Milkify, Inc, Houston, Texas, United States; 3 Rev Bioscience, LLC, Boise, Idaho, United States; 4 Division of Neonatology, Rady Children's Hospital, San Diego, United States; 5 Department of Pediatrics, University of California San Diego School of Medicine, San Diego, California, United States

**Keywords:** freeze-dried mother's own milk, exclusive human milk diet, intestinal atresia, premature infant, intolerance, catch-up growth, case report

## Abstract

**Objective:**

Feeding intolerance and growth failure commonly complicate recovery in infants with complicated intestinal atresia, often requiring prolonged human milk fortification after hospital discharge. Our objective was to describe a novel fortification strategy that enabled an exclusive mother's own milk (MOM) diet during postdischarge fortification in a medically complex infant with feeding intolerance.

**Study Design:**

This case report details the use of freeze-dried mother's own milk (FDMOM) to fortify expressed MOM in a late preterm infant with complicated atresia and congenital shortened bowel to resolve feeding intolerance and weight faltering. Freeze-drying of MOM took place at a commercial facility using SafeDry, a patented contact-free process. FDMOM was used to increase the caloric density of expressed MOM under medical supervision using a targeted fortification approach.

**Results:**

The patient tolerated unfortified MOM but developed severe fussiness, abdominal distention, and increased stooling upon fortification with hypoallergenic formulas. These symptoms resolved within 24 hours of transitioning to FDMOM fortification. Remarkably, the infant went from the 24th percentile for weight-for-age to the 66th percentile within 86 days.

**Conclusion:**

FDMOM fortification may represent a novel, well-tolerated strategy to support growth while maintaining an exclusive MOM diet in infants after complex gastrointestinal surgery and hospital discharge.

## Introduction

Feeding intolerance and impaired growth are common and clinically challenging sequelae in infants with complicated intestinal atresia. 1 These complications frequently necessitate nutritional fortification, with fortification of human milk (HM) often extending into the postdischarge period. 2 HM is widely recognized as the preferred source of nutrition for infants and may be particularly important following gastrointestinal surgery due to its rich content of bioactive components that support mucosal healing and protect against infection, including immunoglobulins, HM oligosaccharides, bioactive proteins, and growth factors. 3 4 However, additional fortification is often required to meet the increased nutritional demands necessary for adequate growth following surgical repair.

HM-derived fortifiers allow infants to remain on an exclusive HM diet when fortification is required. However, current products are not available for use after hospital discharge, creating a gap in nutritional support for medically complex infants after transitioning home. The following case describes the use of freeze-dried mother's own milk (FDMOM) to fortify expressed mother's own milk (MOM) as a strategy to resolve feeding intolerance and weight faltering in an infant with complicated intestinal atresia and congenital shortened bowel due to apple peel deformity of the small intestine. 5

## Case Report

**Fig. 1:**
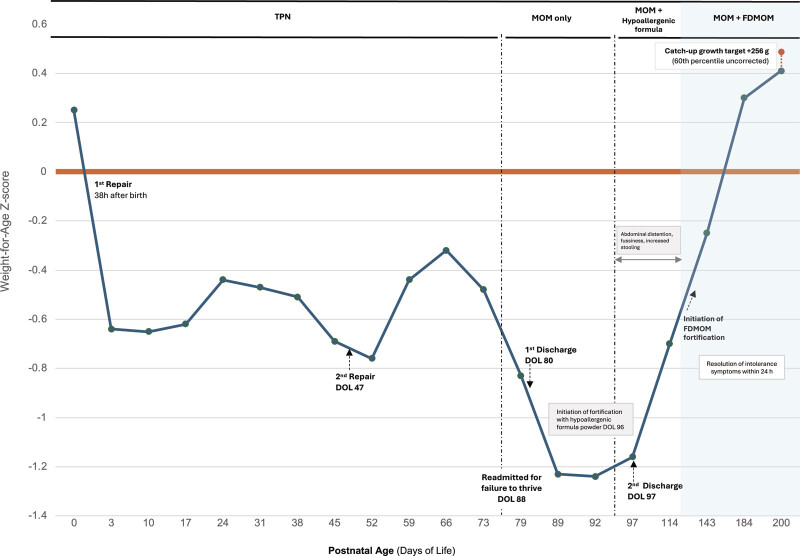
Weight-for-age Z-score (WAZ) trajectory in a late preterm infant with complex intestinal atresia. Following multiple intestinal surgeries, congenital shortened bowel, and prolonged periods of TPN, weight faltering was observed, with WAZ reaching a nadir of −1.24 SD on DOL 92. WAZ were calculated using Fenton 2013 growth references through 50 weeks postmenstrual age and WHO standards using corrected age thereafter. 6 7 During fortification with hypoallergenic formula powders (Nutramigen, Neocate, EleCare), clinically significant feeding intolerance was observed, characterized by abdominal distension, increased stooling, and irritability, which prompted a change in fortification strategy. After initiation of MOM fortified with FDMOM, an upward inflection in growth velocity was observed, with weight reaching 8,119 g (66th percentile; WAZ = 0.41 SD corrected age WHO) by DOL 200, approaching the catch-up growth target (8,375 g; 60th percentile WAZ uncorrected WHO standards). FDMOM, freeze-dried mother's own milk; DOL, day of life; MOM, mother's own milk; NPO, nil per os; TPN, total parenteral nutrition.

A male singleton was delivered vaginally at 35 ^5/7^ weeks' gestation at a tertiary academic referral center with a Level IV neonatal intensive care unit (NICU) following preterm labor in the context of prenatal polyhydramnios with suspicion for bowel obstruction. The center provides comprehensive neonatal surgical and multidisciplinary nutrition support, including established HM-based feeding protocols for medically complex infants. Birth weight was 2.77 kg (weight-for-age 60th percentile, Fenton and Kim 2013 6 ; Fig. 1 ). An initial abdominal radiograph demonstrated a classic double-bubble sign with minimal distal bowel gas. At approximately 38 hours after birth, the infant underwent surgery for duodenal obstruction. Intraoperative findings revealed type IV duodenal atresia with two distinct areas of complete atresia and a markedly enlarged intervening segment, which was dilated to 9 cm (approximately eight times normal) and filled with bile. The distal small bowel also exhibited apple peel deformity and malrotation. The total length of the small bowel was 70 cm. Surgical repair involved resection of atretic segments with reanastomosis incorporating the intervening segment and resection of the apple peel deformity.

Postoperatively, the infant continued to exhibit signs of obstruction. Contrast follow-through showed marked dilation of the second and third segments of the duodenum with no distal contrast passage after 4 hours. The infant returned to surgery on day of life (DOL) 47. The intraoperative findings showed that the duodenal segment remained markedly dilated, and its excessive diameter was thought to impair effective peristalsis. The distal anastomosis had narrowed, consistent with a postoperative stricture. Significant resection of the dilated segment with a stepped tapering procedure was performed with revision of the distal anastomosis to restore luminal patency.

### Nutrition and Intolerance Symptoms

The infant remained on total parenteral nutrition (TPN) for a total of 75 days, receiving hyperalimentation with SMOFlipid and Omegaven. Trophic feeds of MOM were started on DOL 63 and advanced by 10 mL/kg/day as tolerated. During initial hospitalization, the infant received MOM exclusively. He was discharged on DOL 80, weighing 4.68 kg (20th percentile) and taking MOM orally ad libitum.

The infant was readmitted on DOL 88 with dehydration and poor weight gain despite intake of approximately 850 mL of MOM daily. He had ≥15 loose stools daily. Weight upon readmission was 4.73 kg (10th percentile). Beginning on DOL 96, MOM was fortified with an extensively hydrolyzed formula powder to provide an additional 6.8 kcal/ 100 mL (equivalent to +2 kcal/ounce) (Nutramigen with Probiotic LGG, Mead Johnson Nutrition, Chicago, IL). The infant was discharged the following day. However, this fortification was discontinued on DOL 100 due to feeding intolerance, defined as abdominal distention, fussiness, increased stooling, and inability to tolerate feeds exceeding 30 mL. Subsequent fortification strategies using amino acid-based formula powders (Nutricia Neocate, Nutricia North America, Gaithersburg, MD, and Abbott Nutrition EleCare, Abbott Nutrition, Columbus, OH) were also associated with significant abdominal distention, discomfort, and continuation of increased stooling. Parents reported persistent irritability, poor sleep with intervals under 45 minutes, and inability to tolerate feed volumes greater than 30 mL. Neither the cow milk-based hydrolyzed formula nor the amino acid-based formulas were tolerated above +13.5 kcal/100 mL (equivalent to +4 kcal/ounce), the amount deemed necessary to achieve growth goals, assuming the MOM caloric density of 20 kcal/ounce.

Ongoing symptoms prompted a medically supervised trial of FDMOM fortification. Frozen MOM was processed in a certified Good Manufacturing Practices (cGMP) facility using a patented, contactless freeze-drying method (SafeDry, Milkify, Inc., Houston, TX) with subsequent water activity testing (AQUALAB3, Addium, Pullman, WA) to confirm low residual moisture and mitigate microbial risk. Macronutrient composition of both baseline MOM and FDMOM was measured using a MIRIS Human Milk Analyzer (08-02-107, Uppsala, Sweden; Table 1 ). These measurements revealed a baseline MOM caloric density of 63.2 kcal/100 mL (equivalent to 18.7 kcal/ounce) and an FDMOM caloric density of 4.83 kcal/g. A targeted fortification strategy was then developed using these measurements, incorporating both MIRIS-derived nutrient density and water activity data, to calculate the amount of FDMOM required to increase the caloric density of baseline MOM by +20.3 kcal/100 mL (equivalent to +6 kcal/ounce).

**Table 1:** Macronutrient content and energy density of baseline MOM and FDMOM-fortified MOM

Sample	True protein (g/L)	Carbohydrate (g/L)	Fat (g/L)	Energy (kcal/100 mL)
Baseline MOM a	7.0	80.9	29.3	63.2
FDMOM-fortified MOM (+6 kcal/oz) b	7.3	84.3	30.2	83.5

Abbreviations: FDMOM, freeze-dried mother's own milk; MOM, mother's own milk.

a Baseline MOM reflects a 24-hour collection.

b Estimated values based on baseline MOM with 6 kcal/oz added of FDMOM. FDMOM powder composition: Protein 0.059 g/g, carbohydrate 0.695 g/g, fat 0.187 g/g, energy density 4.83 kcal/g.

FDMOM fortification of +20.3 kcal/100 mL (equivalent to +6 kcal/ounce). began on DOL 122, resulting in immediate and sustained improvement in feeding tolerance. Within 24 hours, abdominal distention resolved, and the infant was able to consistently finish full fortified feeds with no further signs of intolerance observed. Parents reported a marked improvement in his overall disposition, and weight-for-age reached the 66th percentile by DOL 200 (WHO reference standard using corrected age 7 ).

## Discussion

To our knowledge, this is the first reported case of targeted postdischarge fortification using FDMOM in an infant with complicated intestinal atresia and congenital shortened bowel due to an apple peel deformity. This late preterm infant was at high risk for growth failure due to two separate surgical repairs, congenitally shortened bowel, prolonged dependence on TPN, and ongoing feeding intolerance. Following discharge, the infant was readmitted with dehydration and poor weight gain. Fortification with hydrolyzed cow milk-based and amino acid-based formulas resulted in significant intolerance. In contrast, intolerance resolved within 24 hours of initiating FDMOM fortification, with subsequent rapid catch-up growth. Weight-for-age plotted with the WHO standards using corrected age reached the 66th percentile within 86 days, demonstrating meaningful improvement in both feeding tolerance and growth following the transition to FDMOM fortification of MOM.

This case underscores key safety considerations for the use of FDMOM as a fortifier. Targeted fortification was possible by measurement of baseline MOM caloric density, which was 18.7 kcal/ounce, below the standard 20 kcal/ounce assumption. Existing data show that freeze-drying preserves the majority of macro- and micronutrients and many bioactive components, 8 with only modest reductions that supplement rather than displace fresh MOM. Our macronutrient analysis supports these findings.

Microbial safety is a primary consideration when evaluating FDMOM for postdischarge fortification in medically fragile infants. In this case, freeze-drying was performed in a cGMP facility using a patented, contact-free process designed to prevent environmental exposure and cross-contamination with HM from other mothers. This closed-system approach offers a substantial safety advantage over traditional freeze-drying methods involving direct contact with equipment and exposure to the surrounding environment. Additional safeguards included environmental pathogen monitoring, water activity testing of the final powder to prevent postdrying microbial growth, and medically supervised fortification with individualized mixing protocols. These measures were essential for the safe implementation of FDMOM fortification in this vulnerable patient.

Some evidence suggests that HM-based fortifiers may be associated with reduced feeding intolerance compared with cow milk-based fortifiers, although findings remain mixed and data on postdischarge strategies are limited. 9 10 11 FDMOM may offer additional advantages by avoiding industrial pasteurization while largely preserving the native composition of MOM. 12

## Conclusion

In summary, this case demonstrates the feasibility and potential clinical benefit of medically supervised, targeted postdischarge fortification using FDMOM in select high-risk infants with weight faltering and feeding intolerance. It highlights a critical gap in postdischarge nutritional care and warrants further investigation.
